# Empathic Accuracy and Cognitive and Affective Empathy in Young Adults With and Without Autism Spectrum Disorder

**DOI:** 10.1007/s10803-021-05093-7

**Published:** 2021-05-29

**Authors:** K. McKenzie, A. Russell, D. Golm, G. Fairchild

**Affiliations:** 1 Centre for Applied Autism Research, Department of Psychology, University of Bath, Claverton Down, Bath, Somerset, BA2 7AY UK; 2 Centre for Innovation in Mental Health, School of Psychology, University of Southampton, Southampton, SO17 1BJ UK; 3 Department of Psychology, University of Bath, Bath, Somerset, BA2 7AY UK

**Keywords:** Autism, ASD, Empathy, Affective empathy, Empathic accuracy, Alexithymia

## Abstract

This study investigated whether young adults with ASD (n = 29) had impairments in Cognitive Empathy (CE), Affective Empathy (AE) or Empathic Accuracy (EA; the ability to track changes in others’ thoughts and feelings) compared to typically-developing individuals (n = 31) using the Empathic Accuracy Task (EAT), which involves watching narrators recollecting emotionally-charged autobiographical events. Participants provided continuous ratings of the narrators’ emotional intensity (indexing EA), labelled the emotions displayed (CE) and reported whether they shared the depicted emotions (AE). The ASD group showed deficits in EA for anger but did not differ from typically-developing participants in CE or AE on the EAT. The ASD group also reported lower CE (Perspective Taking) and AE (Empathic Concern) on the Interpersonal Reactivity Index, a self-report questionnaire.

**Supplementary Information:**

The online version contains supplementary material available at 10.1007/s10803-021-05093-7.

## Introduction

Empathy can be defined as the ability to share others’ feelings or ‘put yourself in their shoes’ (Singer & Lamm, 2009). The literature suggests that empathy is a multi-dimensional concept (Davis, 1980, 1983; Decety, 2015), which includes cognitive empathy (CE), defined as the ability to understand others’ feelings, beliefs and intentions (Baron-Cohen & Wheelwright, 2004; Bos & Stokes, 2019), and affective empathy (AE), which is characterised by “an emotional response in an individual that stems from and parallels the emotional state of another individual” (Smith, 2009, p. 490). Empathy can further be divided into “state empathy”, a psychological state induced by a specific stimulus or situation, and “trait empathy”, a personality tendency which is relatively stable over time (Song et al., 2019).

A wide range of methods have been used to measure CE and AE within the literature, with self-report questionnaire measures being most commonly used. One widely-used questionnaire is the Interpersonal Reactivity Index (IRI) (Davis, 1980), a trait measure of empathy which includes two scales thought to measure AE (‘empathic concern’ and ‘personal distress’), and two which are considered to represent CE (‘fantasy’ and ‘perspective-taking’) (Davis, 1983). However, self-report measures of empathy are subject to several limitations such as social desirability biases, as being empathetic is typically seen as a positive quality. People may also lack insight into their empathic abilities, believing themselves to be more empathic than they really are.

State empathy is usually measured via experimental tasks, such as the Multi-Faceted Empathy Test (MET; Dziobek et al., 2008). The MET requires participants to infer the mental states of people in photographs (measuring CE) and to report their emotional reactions to the pictures (measuring AE). Spontaneous mimicry of another’s emotions is another index of state empathy. For instance, Drimalla et al. (2019) found that one’s tendency to engage in facial mimicry was positively related to AE and CE scores on the MET. Some studies have measured empathy via physiological (e.g., heart rate and skin conductance) responses to emotional stimuli, as these measures are thought to reflect AE (Levenson & Ruef, 1992; Trimmer et al., 2016; Kaplan et al., 1960).

Most existing measures of empathy rely on self-reports of dispositional tendencies or assess subjective or physiological responses to static images (e.g., of sad faces); consequently, they fail to assess the ability to monitor rapidly changing social cues, a skill that is very important in navigating real-life social interactions. The ability to track another person’s (the ‘target’s’) transient thoughts and feelings is known as Empathic Accuracy (EA) (Zaki & Ochsner, 2011; Zaki et al., 2008). Existing measures of EA involve watching videotaped social interactions and assessing a person’s ability to infer the thoughts/feelings of the target (Ickes, 2001), or viewing narrators talking about emotionally-charged experiences and judging the intensity and valence of the target’s emotions (Levenson & Ruef, 1992; Zaki et al., 2009). EA has received less attention in the literature on empathy in ASD compared to CE and AE.

### Empathy in ASD

ASD is characterised by qualitative differences in social communication/interaction and a pattern of repetitive, restricted behaviours, interests and activities (American Psychiatric Association, 2013). People with a diagnosis of ASD^1^ are commonly thought to have difficulties in empathy compared with typically-developing (TD) individuals; as such, ASD has been characterised as an “empathy disorder” (Gillberg, 1992). There are several theories which discuss possible mechanisms underlying empathy deficits in people with ASD. A leading theory of autism suggests that individuals with ASD lack Theory of Mind, meaning they find it difficult to infer the mental states of others (Baron-Cohen, 2000). People with ASD typically perform worse on ‘false-belief’ tasks which measure Theory of Mind by assessing one’s ability to distinguish between events in reality, versus how events may be (incorrectly) represented in another person’s mind (Baron-Cohen et al., 1985). In contrast, the social motivation hypothesis (Chevallier et al., 2012) proposes that individuals with ASD are less socially motivated than TD individuals, which contributes to social impairments such as empathic deficits. Finally, Baron-Cohen’s (2009) ‘Empathising-Systemising’ theory of ASD suggests that people with ASD have a preference towards tasks which involve systemising (e.g., solving maths problems, making lists, fixing bikes, etc.), because systems often change in lawful and predictable ways. However, the theory also indicates that people with ASD struggle with empathy because it is not ‘truth-oriented’; there are no laws which can be applied to understand emotions in others, as different people express emotions in different ways. Using self-report methods, Greenberg et al. (2018) measured the “brain types” (classifying them as either empathising or systemising) of over half a million people, including individuals with a diagnosis of ASD. They found that, when compared to TD participants, the brain types of people with a diagnosis of ASD were balanced in favour of ‘systemising’ at the expense of ‘empathising’ to either a strong or very strong degree.

The Empathy Imbalance Hypothesis (Smith, 2009) provides a more nuanced view on empathy by suggesting that people with a diagnosis of ASD experience “empathic overarousal”, leading them to experience increased distress in response to others’ emotions compared with TD individuals. Consequently, Smith (2009) argues that people with a diagnosis of ASD have heightened AE in comparison to TD individuals, despite showing impairments in CE. An abundance of research has provided evidence for impaired CE in ASD (Baron-Cohen et al., 1997; Dziobek et al., 2008; Baron-Cohen et al., 1997; Ozonoff et al., 1990; Rogers et al., 2007; Zalla et al., 2009), as measured via self-report questionnaires such as the IRI or experimental tasks such as the MET. However, the evidence regarding AE in ASD is mixed, with the majority of studies indicating that people with ASD have either higher or similar levels of AE compared with people without ASD (Dziobek et al., 2008; Murray et al., 2017; Rogers et al., 2007; Song et al., 2019).

Supporting the Empathy Imbalance Hypothesis, in a meta-analysis of 51 studies, Song et al. (2019) found that people with ASD scored higher on IRI Personal Distress, and the AE subscale of the Questionnaire of Cognitive and Affective Empathy (QCAE) (Di Girolamo et al., 2019) compared to TD individuals, suggesting higher trait AE. However, they found that people with ASD scored lower overall on the Empathic Concern subscale of the IRI compared to TD participants. Song et al.’s (2019) findings also indicated that people with ASD performed similarly to TD individuals in experimental tasks measuring AE—most commonly the MET. For example, Dziobek et al. (2008) used the MET in a sample of adults with ASD and found deficits in CE, but not AE, when compared to TD individuals.

More nuanced findings were reported by Mazza et al. (2014), who found that, in comparison to TD participants, individuals with ASD showed deficits in AE (measured by the MET) specifically for negative emotions (such as sadness, anger and disappointment); AE for positive emotions (such as happiness and positive surprise) was unimpaired in the ASD group. Contrary to this, Jankowski and Pfeifer (2021) found that ASD and TD adolescents reported similar levels of AE when watching videos of actors taking part in a singing competition, regardless of whether the emotion was positive or negative. Participants were required to rate the levels of pride and embarrassment felt by the actors and were also asked to rate the levels of pride and embarrassment they themselves felt when watching the videos. CE abilities of participants with ASD (how accurately they rated the emotions felt by the actors) were more strongly influenced by the level of contextual information available. When the context was incongruent with the emotion being displayed (e.g., the contestant performed well but felt embarrassed), participants with ASD were more likely than TD participants to reference contextual information and rate that participants additionally felt proud, although they rated that the participant felt embarrassed to a similar degree.

In contrast, specific impairments in processing negative emotions have been reported in studies measuring CE in ASD populations using facial emotion recognition tasks (e.g., Ashwin et al., 2006). These studies focusing on facial emotion recognition in ASD have suggested that people with a diagnosis of ASD have specific deficits in identifying fear (Howard et al., 2000; Pelphrey et al., 2002), disgust (Golan et al., 2006), sadness (Boraston et al., 2007) and anger from facial expressions (Bal et al., 2010).

There is some, albeit limited, evidence to suggest that individuals with ASD may show atypical neural activity when responding to negative emotions. Krach et al. (2015) measured brain activity whilst participants looked at drawings depicting a protagonist within social scenarios, some of which were neutral, and some of which were likely to induce embarrassment. Compared with TD individuals, participants with ASD showed deficits in AE (measured by the levels of vicarious embarrassment they felt on behalf of the protagonist within the scenarios). Relative to TD controls, the ASD group showed reduced anterior insula and anterior cingulate cortex activity, but greater hippocampus activity, when viewing these scenarios. This suggests that whilst TD individuals are likely to rely on social cues (e.g., facial expressions, body language), people with ASD are more likely to rely on memories of previous social encounters to inform their AE responses.

Further to this, Baron-Cohen et al. (1999) found that individuals with ASD showed significantly less activation in the amygdala overall than TD controls during a task in which participants were required to infer what a person was thinking/feeling by looking at photographs of their eyes. The researchers did not explore whether there was an interaction between group status and emotion on amygdala activity (i.e., whether the ASD individuals showed reduced activation to specific emotions or emotions in general). However, amygdala activation seems to play a particularly important role in the recognition of threat-based emotions such as fear (Adolphs et al., 2005; Baron-Cohen et al., 2000) and anger (Scott et al., 1997). Thus, abnormal amygdala activity in people with a diagnosis of ASD may account for the findings discussed above, which suggest that empathic deficits in ASD might be disproportionately seen for negative emotions; however, further research is required to clarify this. Despite the evidence discussed above which indicates that empathy amongst individuals with ASD may vary across different emotions, few studies have explored this issue whilst measuring multiple components of empathy in parallel. Consequently, the literature may have provided an over-simplified view of empathic abilities in people with a diagnosis of ASD.

Whilst there are a large number of studies examining CE and AE in people with a diagnosis of ASD, primarily using self-report measures, fewer studies have measured empathic accuracy (EA) in ASD. Three studies by the same research group (Demurie et al., 2011; Ponnet et al., 2008; Roeyers et al., 2001) measured EA by asking participants to infer the thoughts and feelings of targets in video-taped social interactions which were either ‘structured’ (playing a board game) or ‘unstructured’ (an unplanned ‘getting acquainted’ conversation). The targets listed the thoughts and feelings they had experienced during the interactions and independent judges assessed how closely the participants’ responses matched the target’s actual thoughts and feelings, yielding an EA score. The authors found that participants with ASD only showed significant impairments when rating the unstructured, but not the structured, social interactions. It should also be noted that these three studies relied on predominantly male samples, which is a common feature of research measuring empathy in ASD populations (Song et al., 2019). This is a key limitation of the existing research, as it means comparatively little is known about empathy in females with ASD.

A recent study by Santiesteban et al. (2021) used an adapted version of the Empathic Accuracy Task (EAT) to measure empathy in 21 adults with ASD and 45 adults without ASD. The EAT assesses AE, CE and EA by measuring participants’ responses to videos of narrators describing autobiographical events. In Santiesteban et al.’s (2021) study, videos depicted either ‘affective’ (sad) or ‘neutral’ events. The narrators provided continuous ratings of how they felt whilst recording the videos. In half of the trials, participants continuously rated the strength of their own emotions whilst watching the video (measuring ‘online’ AE) and, immediately after the video, rated the strength of the actor’s emotions (assessing ‘offline’ CE). In the other half, participants continuously rated the strength of the actor’s emotions during the video (measuring ‘online’ EA), and afterwards gave a single rating reflecting the strength of their own emotions (measuring ‘offline’ AE). Participants with ASD showed deficits in ‘offline’ CE and lower AE compared to TD participants, particularly when viewing ‘affective’ (sad) videos. However, the groups did not significantly differ on the ‘online’ measures of EA and AE. Unfortunately, this study only measured AE, CE and EA in response to sad or neutral events, and did not explore empathic abilities in response to other discrete emotions as anger, happiness or fear.

Santiesteban et al.’s (2021) study also controlled for alexithymia, a trait which is increasingly thought to have an important relationship with empathy (Grynberg et al., 2010). Alexithymia, which is defined as problems in identifying and describing one’s own emotions (Sifneos, 1973), commonly co-occurs with ASD (Hill et al., 2004), and is independently linked to deficits in empathy (Guttman & Laporte, 2002; Prkachin et al., 2009). Research investigating the neural correlates of AE found that differences between people with versus without ASD in anterior insula activity when witnessing a friend experiencing pain were rendered non-significant when alexithymia was accounted for (Bird et al., 2010). More recently, Shah et al. (2019) found that alexithymia was associated with empathy deficits (measured by the Questionnaire of Cognitive and Affective Empathy) in people with autistic traits (but no confirmed diagnosis), but that autistic traits were a more important predictor of empathy than alexithymia. Given the mixed findings in the literature, it is unclear whether empathy deficits in ASD are explained by alexithymia. Thus, we investigated this issue by measuring the impact of alexithymia on different forms of empathy.

### The Current Study

In summary, empathy is a multi-faceted construct with cognitive and affective components which can be expressed and measured at trait and state levels using self-report and experimental methods. Amongst people with a diagnosis of ASD, research has provided consistent evidence of impairments in CE, at both state and trait levels. There is more mixed evidence regarding AE and EA, which appears to be partly accounted for by the variance in measurement methods and lack of granularity in empathy measurement as related to discrete emotions. Furthermore, the generalisability of the existing literature is limited due to the under-representation of females with ASD in studies within this field.

This study therefore aims to improve upon previous research on empathy in ASD by investigating multiple facets of empathy using self-report and experimental methods in a mixed gender sample, while also taking account of alexithymia. We included stimuli depicting happiness, a positive emotion, and a range of negative emotions (anger, sadness and fear) to determine whether deficits in empathy in ASD vary across emotions or are specific to negative emotions. Based on previous research, we predicted that individuals with ASD would show deficits in CE compared to TD participants (Dziobek et al., 2008; Rogers et al., 2007). Although the evidence regarding AE is more mixed, there is no consistent evidence to suggest that AE is impaired in people with ASD. With the exception of trait Empathic Concern (a facet of AE where people with ASD show deficits), the majority of studies using trait measures of empathy indicate that AE is higher in people with ASD compared with TD individuals (Song et al., 2019). Furthermore, experimental studies suggest that people with and without ASD do not differ in state AE (Song et al., 2019). Taking the experimental evidence into account, we predicted that the groups would not differ in AE. It was more difficult to make predictions regarding EA, as this component of empathy has not been widely investigated. Nevertheless, based on studies exploring CE in people with ASD (Jolliffe & Baron-Cohen, 1999; Zalla et al., 2009), and evidence indicating that similar brain regions are involved in CE and EA (Mackes et al., 2018), we hypothesised that participants with ASD would show deficits in EA relative to TD participants. We also predicted that empathic deficits in the ASD group would be partly explained by alexithymia.

## Method

### Design

The study used a between-groups, cross-sectional design.

### Participants

G Power was used to conduct an a priori power analysis, based on Dziobek et al.’s (2008) study which had a similar two-groups design. This suggested that a total of 54 participants (27 per group) was required to detect large effects at an α = 0.05 level of statistical significance and 95% power.

A total of 61 people consented to participate and were divided into two groups; people with a diagnosis of ASD (n = 29) and a control group of TD individuals (n = 32). Participants were matched at a group level in terms of age and gender (ASD Group: 17 males, 12 females; TD Group: 13 Males, 18 females). Participants were all aged 16–25 years and native English speakers. The majority of participants were White British.

Participants were excluded if they had a suspected intellectual disability or current diagnosis of a psychotic disorder. Participants in the ASD group were only included if they had a clinical diagnosis of ASD (confirmed via clinical report or equivalent). Participants in the TD group completed a brief version of the Autism Quotient (AQ-10) (Baron-Cohen et al., 2001) to screen for traits of ASD; they were excluded if they scored above the screening cut-off (≥ 6). One participant was excluded on this basis.

### Recruitment

Participants were recruited via the University of Bath’s Research Community Participation Pool, the Centre for Applied Autism Research database and local educational programmes, such as a summer school for autistic students considering going to university. Poster advertisements were also placed in several university departments. Participants were reimbursed five pounds for taking part.

### Self-Report Measures

The IRI (Davis, 1980) was used to measure trait empathy. This is a 28-item self-report measure which consists of four subscales; ‘Empathic Concern’ and ‘Personal Distress’ (thought to measure AE); ‘Perspective Taking’ and ‘Fantasy’, (thought to measure CE). The IRI has adequate internal consistency with alpha coefficients ranging from 0.68 to 0.79 (Baldner & McGinley, 2014; Davis, 1980). The IRI has previously been used to measure trait empathy in people with ASD across numerous studies (Song et al., 2019).

The Toronto Alexithymia Scale (TAS-20) (Taylor et al., 1985), a 20-item self-report questionnaire, was used to measure trait alexithymia. Research indicates that this scale has moderate to high reliability when used in ASD populations (Cronbach’s α = 0.86) (Samson et al., 2012).

The presence of ASD traits in the TD group were measured using a brief version of the Autism Quotient (AQ-10). The AQ-10 has good sensitivity and specificity (Allison et al., 2012). Booth et al. (2013) found that the AQ-10 had an area under the curve of 90.3%, indicating excellent predictive validity.

Following data collection, the researchers conducted reliability analyses which showed that both the IRI (α = 0.845) and TAS-20 (α = 0.851) fell into the ‘good’ range for reliability.

## Experimental Empathy Measure: The Empathic Accuracy Task (EAT).

CE, AE and EA were measured using a modified version of the EAT (Mackes et al., 2018; Zaki et al., 2009), which has previously been used to explore EA in people with schizophrenia (Lee et al., 2011; van Donkersgoed et al., 2019) and adolescents with conduct disorder (Martin-Key et al., 2017, 2020).

Using a laptop, participants watched ten video clips of narrators describing autobiographical experiences in which they had felt discrete primary emotions. Please see Supplementary Material for an overview of the events described in each clip. Two narrators (one male, one female, both White British) depicted five emotions each (anger, happiness, sadness, fear and no emotion). Seven participants with ASD completed the task using clips which were filmed for a previous study (Mackes et al., 2018). However, due to ethical concerns related to the content of one clip (the female narrator described an incident of domestic violence), the researchers filmed new video clips with a different female narrator. The five original female clips were replaced with five clips using the new female narrator; however, the original male narrators’ clips were used throughout the study. The events described in the new clips were filmed closely following the procedure used by Mackes et al. (2018) (although there were some minor differences which are highlighted in Supplementary Material). All of the clips were rated as five or above in emotional intensity by the narrator (rated on a 1–9 scale, with 9 being the highest score). The narrator was also prohibited from directly mentioning the emotion they were depicting whilst describing the event (e.g., ‘I felt *angry*’). After filming all five clips, the narrator rated the intensity of the emotion they experienced whilst describing each event. Further information about the filming procedure can be found in our Supplementary Materials.

All participants in the TD group, and the remaining 22 participants in the ASD viewed the clips with the original male narrator and the new female narrator (they did not view the original female clips).

**Fig. 1:**
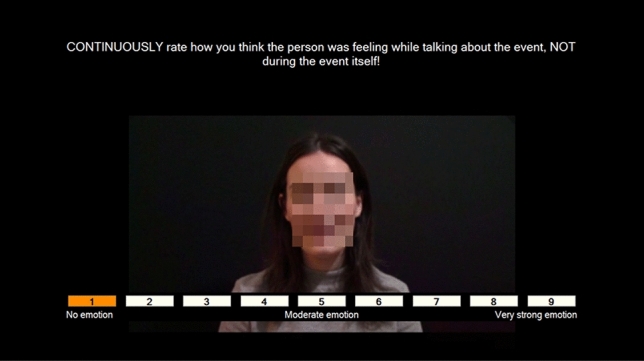
A screenshot from the Empathic Accuracy Task. The narrator’s face has been pixelated to protect their identity but was visible in the actual task

The ten clips were arranged into two playlists (A and B), each presenting the clips in different orders. To reduce order effects, the playlists were counterbalanced across participants (and groups). Whilst watching the clips, participants used the arrow keys on the keyboard to continuously rate the intensity of the narrator’s emotions on a scale from 1 (no emotion) to 9 (very strong emotion) (Fig. 1). The correlation between the narrators’ ratings of their own clips (how they felt while talking about the event) and the participant’s ratings of the narrator’s feelings yielded the measure of EA.

After watching each clip, participants were asked to select the predominant emotion displayed from a list of six primary emotions (measuring CE). Finally, participants labelled the emotion they themselves experienced whilst watching the clip (there was also an option of ‘No Emotion’), providing a binary CE score (correctly or incorrectly identifying the narrator’s emotion). The extent to which their emotion matched the predominant emotion displayed by the narrator provided a binary AE score (the participant either experienced the same emotion as the narrator, or a different emotion/no emotion). As there were two trials per emotion for both CE and AE, participants could score either 0% (incorrect on both trials), 50% (correct on one trial) or 100% for each emotion (correct on both trials).

As AE abilities may be dependent on CE abilities (Coll et al., 2017), the researchers also calculated a second measure of AE (AE2) by assessing whether the emotion the participant was experiencing matched the emotion that they *thought* the actor was experiencing.

The EAT was initially piloted with an individual with a diagnosis of ASD. Their feedback was used to refine the final experimental protocol.

### Governance and Ethical Issues

Ethical approval was gained from the University of Bath’s Psychology Research Ethics Committee. During the debrief, the experimenter asked the participant how they were feeling and administered a mood repair task if the participant felt distressed.

### Procedure

Participants completed the self-report measures prior to the EAT (the AQ-10 was completed by the TD group only). Participants were debriefed after the session, which lasted up to one hour. No participants reported feeling distressed following the EAT and thus the mood repair task was not used.

### Analysis

Demographic variables were compared across groups using independent *t* tests and Chi-Square tests. Correlational analyses were run to test for associations between the IRI scores and EA, CE and AE scores on the EAT. Criterion validity was assessed by correlating IRI scores with EAT Scores (see Supplementary Material).

The IRI and EAT data were not normally distributed, therefore Mann–Whitney *U* tests were used to compare IRI scores between groups, applying a Holm-Bonferroni correction (Holm, 1979) to control for familywise error rates. The EAT data were analysed using the procedure outlined by Martin-Key et al. (2017). The emotional intensity ratings provided by each participant were separated into two second bins. Correlations between these ratings and the narrators’ own ratings were then determined. These correlations were transformed using Fisher’s Z, resulting in one EA score per clip. Average total EA scores and EA scores for each emotion were then calculated for each participant. Mann–Whitney *U* tests were used to compare groups in terms of EA, AE and CE scores (total scores and scores for individual emotions). The same method was used to explore whether the ASD group’s EA, AE and CE scores (total scores and scores for each emotion) differed depending on whether they watched the old or new clips.

Preliminary analysis of the EAT data revealed that participants (n = 7, ASD Group) who watched the ‘old’ film clips (the original male and female narrators, before the female video clips were re-filmed) had significantly lower EA scores compared with those who watched the ‘new’ clips (with the original male narrator and new female narrator) [n = 22 (ASD Group), n = 31 (TD Group)], suggesting that it may not be valid to combine the data from participants who watched both types of clips (old and new). The seven participants who watched the old clips were therefore excluded from further analyses comparing the groups’ EA scores, meaning the ASD Group’s sample size for the EA analyses is 22 rather than 29. Analyses of EA data including the seven participants who watched the original clips is available in the Supplementary Material. As there were no significant differences in the CE or AE scores of participants who watched the old film clips versus those who watched the new clips, all (n = 59) participants were included in the CE and AE analyses.

To investigate whether alexithymia was associated with empathy in the ASD group, total TAS-20 scores were correlated with IRI and EAT scores for the ASD group only (n = 29 for IRI, AE and CE scores, n = 22 for EA Scores).

In addition to the 7 participants who were excluded from analyses of the EAT on the basis of viewing the original film clips, one additional TD participant was excluded from the analyses of Total EA scores and EA for Fear. This is because they did not change the default emotional intensity rating for the frightened clips, meaning their EA score for this clip could not be calculated. Consequently, it was not possible to determine their Total EA score. Additionally, EAT data was missing for one TD participant due to technical issues. Therefore for the EAT analyses, the final sample was n = 29 (ASD Group) and n = 30 (TD Group) for the CE and AE scores, and n = 22 (ASD Group) and n = 30 (TD Group) for the EA scores.

## Results

### Demographic Characteristics

There were no differences between the ASD and TD groups in age [*t*(58) = 1.11, *p* = 0.272], gender ($\chi^{2}$ (1) = 1.669, *p* = 0.196) or highest education qualification achieved ($\chi^{2}$ (1) = 4.518, *p* = 0.211) (see Table 1). Significantly more participants in the ASD group than the TD group reported current mental health difficulties, $\chi^{2}$ (2) = 10.043, *p* = 0.007.

**Table 1:** Demographic and clinical characteristics of the sample

	ASD group (n = 29)	TD group (n = 31)	*p* value
Age	18.31 (1.65)	17.81 (1.85)	0.272
Gender			0.196
Male	17 (58.6)	13 (41.9)	
Female	12 (41.4)	18 (58.1)	
Current mental health difficulties^a^			0.007*
Anxiety disorder	11 (37.9)	4 (12.9)	0.025*
Social anxiety	7 (24.1)	0	0.004*
Depression	3 (10.3)	0	0.107
Eating disorder	1 (3.4)	0	0.475
Borderline PD^b^	1 (3.4)	0	0.483
Prefer not to say	3 (10.3)	1 (3.2)	0.346
Highest educational qualification			0.211
None	0	0	
GCSEs	14 (48.3)	21 (67.7)	
A-Levels	13 (44.8)	7 (22.6)	
University first degree	2 (6.9)	1 (3.2)	
Prefer not to say	0	1 (3.2)	
Age of autism diagnosis			
Before age 5	8 (27.6)		
5–11	9 (31)		
12–17	10 (34.5)		
18 or over	2 (6.9)		

Mean (Standard Deviation) is given for age, all other values show numbers of participants with percentages shown in paratheses

^*^p < 0.05

^a^Participants could have more than one mental health difficulty

^b^*PD* Personality Disorder

### Trait Empathy (IRI Scores)

#### Cognitive Empathy.

The ASD group scored significantly lower than the TD group on the Perspective Taking subscale, *U* = 214.5, *z* = − 3.484, *p* < 0.001, *r* = − 0.45 (see Table 2) but not on the Fantasy subscale of the IRI (*U* = 320, *z* = − 1.921, *p* = 0.055). Although studies of typically-developing adults have reported gender differences in self-report measures of empathy (Baez et al., 2017), scores on the Perspective Taking subscale did not significantly differ by gender; this was true when collapsing across groups (*U* = 341 *z* = − 1.62*, p* = 0.106) and when analysing the ASD Group only (*U* = 101 *z* = − 0.045*, p* = 0.983).

**Table 2:** Self-reported trait empathy scores as measured using the Interpersonal Reactivity Index (IRI)

	ASD group (n = 29)	TD group (n = 31)	*p* value	Effect size (*r*)
Total score	60.83 (14.43)	67.61 (12.45)	0.024	− 0.29
Cognitive empathy				
Perspective taking	13.59 (4.59)	18.29 (5.05)	< 0.001**	− 0.45
Fantasy	15.45 (5.75)	18.16 (4.73)	0.055	− 0.25
Affective empathy				
Empathic concern	16.66 (4.87)	19.52 (5.03)	0.012*	− 0.32
Personal distress	15.14 (5.88)	11.65 (5.21)	0.029	− 0.28

As multiple comparisons were performed, *p* values were adjusted using the Holm-Bonferroni method

* < 0.0125, ** < 0.01; Values are given as Means (Standard Deviations)

#### Affective Empathy.

The ASD group scored significantly lower than the TD group on the Empathic Concern subscale, *U* = 280.5, z = − 2.51, *p* = 0.012, *r* = − 0.32 (see Table 2). The ASD group also scored higher on Personal Distress than the TD group, but not significantly so following correction for multiple comparisons (*U* = 302, *z* = − 2.186, *p* = 0.029) (using an adjusted alpha of *p* < 0.025).

### Empathy Accuracy Task (EAT) Results

#### Cognitive Empathy.

No significant group differences were found for Total CE (*U* = 335, *z* = − 1.563, *p* = 0.118), or CE for Happiness (*U* = 415, *z* = − 0.449, *p* = 0.654), Sadness (*U* = 422.5, *z* = − 0.304, *p* = 0.761), Anger (*U* = 359.5, *z* = − 1.295, *p* = 0.195), Fear (*U* = 385.5*, z* = − 0.805, *p* = 0.421) or Neutral clips (*U* = 400, *z* = − 0.760, *p* = 0.448) (Table 3).

**Table 3:** Empathic accuracy task (EAT) scores by group and group comparisons

	ASD group	TD group	*p* value	Effect size (*r*)
	(n = 29)	(n = 30)		
Cognitive empathy				
Total	78.28 (15.13)	83.67 (13.26)	0.118	− 0.20
Happiness	87.93 (25.55)	91.67 (18.95)	0.654	− 0.06
Sadness	93.10 (17.55)	91.67 (18.95)	0.761	− 0.04
Anger	68.97 (31.19)	78.33 (31.30)	0.195	− 0.17
Fear	55.17 (40.85)	63.33 (41.38)	0.421	− 0.10
Neutral	86.21 (26.38)	91.67 (18.95)	0.448	− 0.10
Affective empathy				
Total	52.07 (21.28)	62.67 (21.16)	0.060	− 0.24
Happiness	53.45 (39.94)	71.67 (33.94)	0.073	− 0.23
Sadness	75.86 (31.68)	80.00 (38.51)	0.307	− 0.13
Anger	20.69 (34.11)	40.00 (40.26)	0.046	− 0.26
Fear	20.69 (31.39)	23.33 (38.80)	0.949	− .001

	(n = 22^a^)	(n = 30)		
Empathic accuracy (New clips only)				
Total	1.13 (0.22)	1.20 (0.13)	0.267	− 0.16
Happiness	1.12 (0.28)	1.05 (0.18)	0.868	− 0.02
Sadness	1.30 (038)	1.39 (0.21)	0.415	− 0.11
Anger	1.03 (0.24)	1.20 (0.19)	0.004*	− 0.40
Fear	1.12 (0.30)	1.16 (0.24)	0.509	− 0.09

*p* values were adjusted for multiple comparisons using the Holm-Bonferroni method

*p < 0*.*01; Values are given as Mean (Standard Deviation)

^a^n = 21 for EA Fear and Total EA due to the exclusion of one participant in the ASD Group

#### Affective Empathy.

Correcting for multiple comparisons, there were no significant group differences in Total AE (*U* = 312.5, *z* = − 1.88, *p* = 0.060) or AE for Happiness (*U* = 325.5, *z* = − 1.794, *p* = 0.073), Sadness (*U* = 379.5, *z* = − 1.022, *p* = 0.307), Anger (*U* = 317.5*, z* = − 1.991, *p* = 0.046, *r* = − 0.259) or Fear (*U* = 431.5, *z* = − 0.064*, p* = 0.949). When AE2 scores were used, similar (non-significant) results were obtained, indicating that AE did not differ between the groups even if participants were not penalised for misidentifying the emotion at the first step.

#### Empathic Accuracy.

**Fig. 2:**
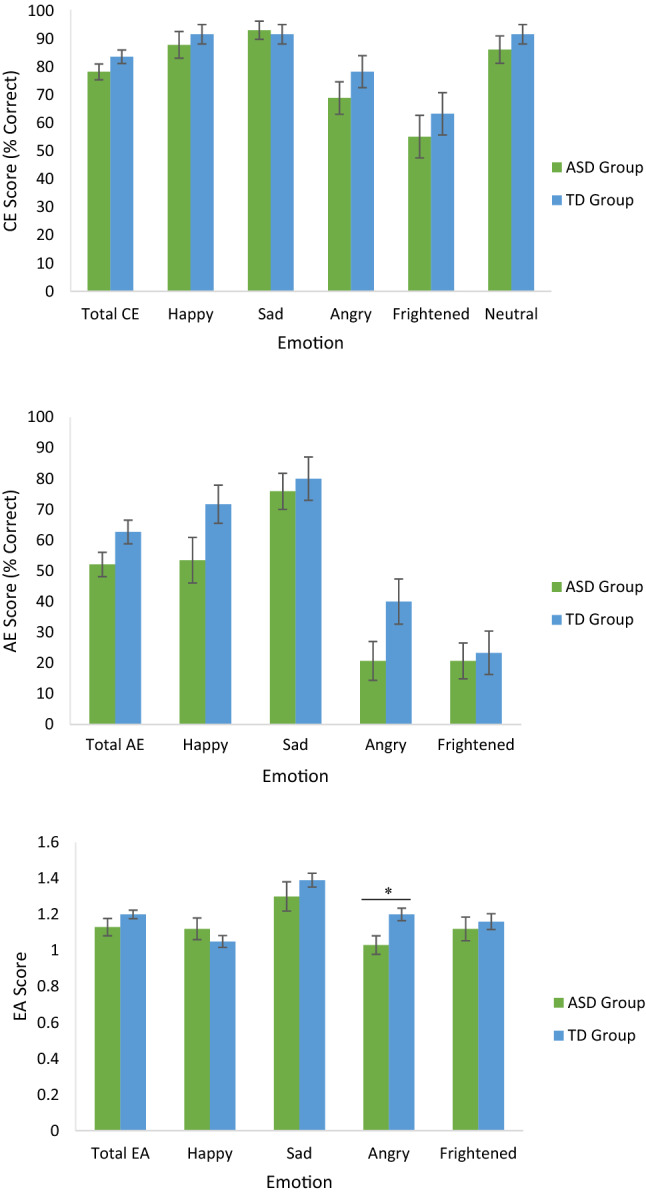
Mean cognitive empathy, affective empathy and empathic accuracy scores by group (error bars show standard error; asterisk depicts significant *p* value following correction for multiple comparisons)

The ASD group scored significantly lower in EA for Anger compared with the TD group, *U* = 174, z = − 2.89, *p* = 0.004, *r* = − 0.40 (Table 3). However, the ASD and TD groups did not significantly differ in Total EA score (*U* = 257, *z* = − 1.11, *p* = 0.267), and EA for Sadness (*U* = 286, *z* = − 0.815, *p* = 0.415). Fear (*U* = 280.5, *z* = 0.66, *p* = 0.509) or Happiness (*U* = 321, *z* = − 0.167, *p* = 0.868) (see Fig. 2).

### Relationship Between Alexithymia and Empathy in the ASD Group

In terms of trait CE, alexithymia scores were not significantly correlated with IRI Perspective Taking, *r*(28) = − 0.075, *p* = 0.699, or Fantasy, r(28) = − 0.074, *p* = 0.704. There was a marginally significant *positive* correlation between alexithymia scores and CE for anger [*r*(28) = 0.366, *p* = 0.051] in the ASD group. However, alexithymia did not correlate significantly with any other experimental CE scores. For trait AE, there was a significant positive correlation between alexithymia and IRI Personal Distress subscale of the IRI in the ASD group, *r*(28) = 0.484, *p* = 0.008 (see Supplementary Material for a scatterplot). However, the correlation between alexithymia and IRI Empathic Concern was non-significant, *r*(28) = − 0.327, *p* = 0.083, as were the correlations between alexithymia and all experimental AE measures. EA Scores for Anger were not significantly correlated with alexithymia scores, *r*(21) = − 0.041, *p* = 0.858. Similarly, no significant correlations were found between alexithymia and EA scores for the other emotions. Please see Supplementary Materials for details of the non-significant findings *(p* values ranged from 0.057 to 0.980; *r* values ranged from − 0.026 to 0.366). This pattern of correlations suggests that alexithymia does not explain the deficits in trait empathy or EA for anger observed in the ASD group.

### Relationship Between Alexithymia and Empathy in the TD Group

No significant correlations were found between alexithymia and any of the trait or experimental empathy measures in the TD Group. Please see Supplementary Material for details of the correlations between Alexithymia and EAT scores in the TD Group (*p* values ranged from 0.058 to 0.962; r values ranged from − 0.009 to 0.306).

## Discussion

The current study investigated cognitive empathy (CE) and affective empathy (AE) in young adults with a diagnosis of ASD and is one of the first studies to investigate autistic participants’ ability to continuously track changes in emotional intensity (i.e., Empathic Accuracy; EA), using dynamic stimuli involving auditory, visual and verbal information. Participants in the ASD group showed some deficits in trait CE, but they did not differ from the TD Group in terms of experimental CE. Similarly, the ASD group reported some impairments in trait AE, but did not show deficits in experimental AE relative to the TD group. The ASD group showed reduced EA for anger relative to the TD participants, but otherwise showed no significant differences in EA, although a slightly reduced sample size for the EA analysis may have decreased the level of statistical power, affecting the likelihood of detecting group differences. Neither the deficit in EA for anger nor the impairments in trait CE and AE in the ASD group were explained by alexithymia.

In line with previous findings (Bird et al., 2010; Rogers et al., 2007), the ASD group scored significantly lower on trait CE as measured by the Perspective Taking subscale of the IRI. However, contrary to past research (Dziobek et al., 2008; Mul et al., 2018), the ASD group performed similarly to the TD group on the CE component of the EAT (although small differences for anger were observed). This finding runs counter to previous results suggesting that people with ASD perform significantly worse in experimental tasks measuring experimental CE compared to TD individuals (Dziobek et al., 2008; Lockwood et al., 2013; Mul et al., 2018). However, the EAT differs from the majority of empathy measures/tasks, as it uses video clips of people recollecting real-life autobiographical experiences. It is possible that using such videos increases the availability and richness of cues signalling emotions, making it easier for participants to ‘read’ the narrators’ emotions and detect changes in emotional intensity. Although this arguably provides a more realistic measure of CE compared to tasks like the MET which use photographs, the sensitivity of this element of the EAT could be improved, particularly as there were only two emotions per clip; however, previous studies have found deficits in CE in other clinical groups, such as adolescents with conduct disorder (Martin-Key et al., 2017), indicating that the EAT is sensitive enough to detect between-group differences in cognitive empathy. Future studies could include a larger number of clips depicting high and low intensity examples of each emotion to examine this issue in more detail (as was done by Lee et al., 2011 in their study investigating empathy in patients with schizophrenia).

As for AE, participants with ASD reported having significantly lower levels of Empathic Concern on the IRI compared with TD participants. This is consistent with Song et al.’s (2019) meta-analysis and research showing that children with ASD are less likely to display concern towards an experimenter in distress (Hobson et al., 2009). The ASD group also showed slightly higher scores on the IRI Personal Distress subscale compared to the TD group; however, contrary to previous findings (Song et al., 2019), the differences between the groups were not significant.

There were no significant differences between the ASD and TD groups in AE, as measured within the EAT. This is in line with previous research which measured experimental AE and CE (Dziobek et al., 2008; Mul et al., 2018). The current findings therefore appear to contradict the theory that people with ASD have an “empathy imbalance”, with disproportionately high levels of AE and low levels of CE (Smith, 2009). Although not a statistically significant finding, participants with ASD tended to show lower levels of experimental AE in response to anger, which is consistent with the findings obtained for EA. Similarly, Mazza et al. (2014) found that participants with ASD performed worse in the AE component of the MET when responding to negative emotions (e.g., anger, sadness and disappointment).

The finding that the ASD Group showed an EA deficit for anger is in line with the results of a study by Bal et al. (2010), who found that, compared to TD children, children with ASD showed deficits in detecting angry, but not fearful, facial expressions. They concluded that people with ASD require more contextual cues to detect anger in comparison to TD people. As anger is arguably a less socially acceptable emotion to express than sadness, happiness or fear, narrators in the EAT might have found it more difficult to bring an angry memory to mind, or may have struggled to directly express anger during the filming process. This means that the emotion displayed by the narrator may have appeared less congruent with the memory they were describing and may have been less easy to ‘read’. However, individuals in the TD Group did not show a trend towards lower EA anger scores, indicating that they were unaffected by this potential incongruency. When investigating CE, Jankowski and Pfeifer (2021) found that people with ASD are more likely to show differences in CE when the context was incongruent with the emotion being displayed, whereas the CE abilities of TD individuals were less reliant on whether context was congruent. The researchers argued that processing these incongruent scenarios placed greater demand on perspective-taking abilities, i.e., participants with ASD tended to infer the emotional state based on the description provided to a greater degree than the TD individuals, who predominantly inferred the emotional state being conveyed by the actor. The findings of the current study and previous research (Baron-Cohen, 2000; Song et al., 2019) suggest that people with ASD show deficits in perspective-taking. Thus, the ASD group in the current study may have struggled to take the perspective of the narrators in the angry clips, leading to lower EA scores. However, further research is required which specifically explores how contextual information affects EA in response to a range of discrete emotions including anger.

It is also possible that people with ASD perceive angry faces to be particularly threatening. This may be because anger is more likely to result in physical harm, in comparison with other emotions. Anger may also be perceived to be a particularly unpredictable emotion in terms of how it manifests; unpredictability can be particularly anxiety-provoking for people with ASD (Robertson et al., 2018). The ideas above are supported by the findings of Garcia-Blanco et al. (2017) who found that children with ASD showed an attentional bias away from pictures of angry faces (but not sad or happy faces), when compared with TD children. They linked their findings to evidence that people with ASD experience overstimulation in the amygdala when processing threatening emotions like anger and fear (Kleinhans et al., 2010), and proposed that the attentional bias helped participants with ASD to regulate their personal distress. This attentional bias may be replicated in real life scenarios, making it even more difficult for people with ASD to read social cues. It is possible that this contributes to deficits in EA for anger, although this warrants further exploration.

The ASD group did not show deficits in EA for other emotions; this is contrary to the findings of previous research (Demurie et al., 2011; Ponnet et al., 2008; Roeyers et al., 2001) which indicates that people with ASD show deficits in EA when responding to ‘unstructured’ social scenarios (although these studies did not measure responses to discrete emotions). This may be linked to a lack of statistical power in the current study, given that data from seven participants were excluded from the EA analyses. These contrasting findings may also be accounted for by the differing approaches to measuring EA; these previous studies measured responses to social scenarios which were manipulated by the researcher, whereas the EAT measures participants’ responses to video clips depicting real-life experiences. Furthermore, the EAT assessed the participants’ ability to track changes in emotional intensity from moment to moment, whereas the aforementioned studies measured ability to identify targets’ thoughts and feelings retrospectively, with no time limit.

In the most comparable study in the literature, Santiesteban et al. (2021) measured EA using an adapted version of the EAT. They found that participants with ASD were unimpaired in EA, which is largely in line with the results of the current study (although they only examined responses to sad emotions). Some researchers (Schilbach, 2014; Santiesteban et al., 2021) have proposed that ‘online’ empathy tasks (which require participants to respond rapidly to changes in emotions), such as the EA component of the EAT, may place less demand on Theory of Mind abilities compared to tasks which assess ‘offline’ or retrospective social cognition. Theory of Mind (ToM) is a reflective process which allows us to infer the mental states (emotions, beliefs, intentions) of ourselves and others, and is thought to be impaired in ASD (Baron-Cohen, 2000). It is closely linked to CE and the two terms are often used interchangeably.

Consistent with this, participants with ASD in the current study showed deficits in Perspective Taking measured by the IRI, which can be classed as an ‘offline’ measure as it retrospectively assesses CE. However, they did not show deficits in CE on the EAT (also an ‘offline’ measure, as it was measured after, rather than during, the presentation of the stimuli). Conversely, participants with ASD in Santiesteban et al.’s (2021) study were impaired on an offline measure of experimental CE; however, their CE measure assessed participants’ ability to detect changes in the intensity of the target’s emotions (on a 0–10 rating scale) rather than identifying the emotion displayed. It is possible that assessing changes in emotional intensity places more demands on ToM compared to emotion identification; however, the cognitive processes underlying online versus offline empathic processes require further research.

In the current study, we found that empathic deficits in people with ASD could not be explained by alexithymia. Furthermore, contrary to our hypothesis and previous research showing an association between alexithymia and impaired empathy [using facial emotion recognition tasks (Prkachin et al., 2009) and empathic brain responses (Bird et al., 2010)], we actually found a positive (albeit weak) relationship between alexithymia and some aspects of empathy in the ASD Group (Personal Distress on the IRI and CE for anger). The positive association between alexithymia and personal distress has also been found in previous research (Guttman & Laporte, 2002; Moriguchi et al., 2007). It is possible that people with high levels of alexithymia find it difficult to process how another person’s distress is impacting them emotionally, leading them to feel overwhelmed. Furthermore, personal distress is thought to be an empathic ability which is represented at a less abstract level (Moriguchi et al., 2007), which may explain why its relationship with alexithymia differs in comparison to other facets of empathy. However, although the current findings provide preliminary evidence that alexithymia may play a role in CE for anger and Personal Distress, this is an area which warrants further research.

### Implications and Future Directions

The contrast between the findings of the trait and experimental empathy measures may be explained by individuals with ASD having a negative (or more realistic) view of their empathic abilities, leading them to report lower levels of empathy on the IRI. Alternatively, these differences may be linked to the sensitivity of the CE and AE components of the EAT, which was restricted in comparison to the IRI. The nuanced findings of this study and apparent discrepancies between different measures of empathy reinforce the conclusion that empathy is a complex construct, which is highly dependent on the context in which it is measured. By measuring EA and using a range of discrete emotions, the EAT has improved upon previous experimental measures in its ability to capture this complexity; however, further work is required to develop more dynamic measures of empathy. It may also be informative to use the EAT in a sample of children or adolescents with and without ASD, to explore whether differences in empathy are more pronounced at a younger age (and the specificity of the deficits in EA for anger observed here).

In addition, more research focusing on CE, EA and AE in people without ASD across varying contexts may be helpful in challenging the common narratives surrounding ASD and empathy. For instance, the ‘double empathy’ hypothesis (Milton, 2012) is based on research which indicates that people without ASD also show difficulties in empathy when responding to emotions displayed by people with ASD (Sasson et al., 2017; Sheppard et al., 2016). It would be interesting to explore this in relation to EA, AE and CE, to examine whether similar empathic deficits are observed when TD individuals view autobiographical experiences recounted by those with ASD. This could help in challenging the simplistic view that people with ASD have impaired empathy, and in promoting a more nuanced view, that people with differing brain types and different experiences of the world may, in some scenarios, have difficulties empathising with each other (Milton, 2012).

The finding that the ASD group showed deficits in EA when responding to anger has implications for social interactions, e.g., people with ASD may find it harder to detect changes in anger displayed by others; consequently, they may respond inappropriately, inadvertently causing the person to become more irate. This may put them at higher risk of experiencing interpersonal conflict, physical harm and lead to difficulties maintaining relationships. In order to understand why people with ASD show this deficit, further research examining brain activity in people with and without ASD during EAT performance is required. Furthermore, as people with ASD may require more information to detect anger in others, it may be helpful to develop interventions to enhance EA in this population.

### Strengths and Limitations

One of the main strengths of this study is that it employs a more naturalistic and dynamic measure of empathy than the tasks that have been used previously. Furthermore, assessing empathy using both an experimental task and a validated self-report questionnaire allowed for a richer exploration of empathy as a multidimensional construct. The measurement of variables which have been shown to influence empathy (such as alexithymia and mental health problems) is a further strength. Possible confounding variables were also addressed by matching groups for age and gender; this is also one of the first experimental studies on empathy to include a well-balanced sample of male and female participants with ASD.

A limitation of the study is that a lack of statistical power may have reduced the likelihood of detecting statistically significant group differences, particularly for EA. The power calculation was performed using data from a study which used a different experimental measure of empathy, which may have implications for the validity of the power calculation. If a larger sample had been used, it is possible that more widespread empathy deficits would have been found within the ASD Group. A larger sample would have allowed for more meaningful exploration of gender differences; with greater statistical power, we could have adopted a four-group design and investigated whether empathy differed according to diagnostic group and gender. Unfortunately, the moderate sample size in the present study means that we would have been underpowered to detect interactions between gender and group on empathic accuracy, CE or AE. This is nevertheless an important area for future research given prior research reporting gender differences in the relationship between empathy and other clinical disorders such as Conduct Disorder (Martin-Key et al., 2020).

Furthermore, the CE and AE scores, in which participants are categorised as either ‘right’ or ‘wrong’, may result in an overly-simplified measurement of these constructs. More nuanced measures of CE and AE could be developed in the future; for example, by asking participants to rate the intensity of the narrators’ emotion on a nine-point scale (CE). A further limitation is that the authors failed to systematically collect information about the participants’ ethnicity. This information would have been useful given that all of the narrators in the EAT were White British.

### Conclusions

The current findings suggest that people with a diagnosis of ASD show selective deficits in EA for anger, but no deficits in other aspects of empathy (AE or CE) on the experimental task. In contrast, some deficits were found in trait CE and AE (as assessed using the IRI) in the ASD group, whereas there was a non-significant tendency toward the ASD participants reporting higher levels of personal distress (a measure of AE). These findings challenge the notion that ASD is an ‘empathy disorder’ as they indicate that people with ASD are capable of recognising dynamic emotions and the emotional states of others. Furthermore, the findings highlight the need for further research exploring empathic responses to anger in ASD and the importance of assessing empathy to discrete emotions in future studies in this field.

## Supplementary Information

Below is the link to the electronic supplementary material.

Supplementary file1 (DOCX 46 KB)

## Footnotes

1 A large scale study by Kenny et al. (2016) indicated that there is no single preferred term to describe people with a diagnosis of ASD. Furthermore, there is debate regarding the use of person-first or identity-first language when describing ASD (Vivanti, 2020). Considering this, the term ‘people with a diagnosis of ASD’ will be used throughout this paper.
