# Supplementary material for: Empathic Accuracy and Cognitive and Affective Empathy in Young Adults With and Without Autism Spectrum Disorder
Source: J Autism Dev Disord. 2021 May 29;52(5):2004–18. doi: 10.1007/s10803-021-05093-7 (PMC9021079; doi:10.1007/s10803-021-05093-7)
Supplement: Supplementary file 1 — Supplementary file1 (DOCX 46 KB) [file 10803_2021_5093_MOESM1_ESM.docx]

**Supplementary Method**

**Filming Procedure for EAT Clips**

The researcher followed the protocol which was used to film the original video clips (Mackes et al., 2018), with some minor exceptions which will be highlighted below. The narrator was firstly asked to write a short paragraph about four different autobiographical events in which she had experienced one of the four emotions being studied (happy, sad, angry and frightened). She was also asked to rate the intensity of each emotion from one to nine. As per the original protocol, the researcher ensured that each emotion was rated at a minimum of five.

Before filming each emotion, the researcher read out a script to help to further elicit the emotion linked with the event. The narrator was then filmed against a backdrop, in a room with a daylight bulb, with just her head and shoulders in view. She was asked to describe the events she had written down earlier, one at a time. In addition to the four primary emotions, the narrator was asked to film a ‘neutral’ clip in which she described her bedroom.

The narrator was given approximately 90 seconds to discuss each event. The order of the events she spoke about was pseudorandomised, so that positive and negative emotions were intermixed. The narrator was also given a ten-minute break between filming each clip, in order for the elicited emotions to be processed and washed-out before filming the next clip (this was not the case during the original filming procedure (Mackes et al., 2018)).

Immediately after filming the last clip, the video clips were transferred onto a computer and integrated into an amended version of the EAT.

In line with the procedure used in the development of the first version of the EAT (Zaki et al., 2008), the narrator watched each film clip, and was instructed to continuously rate the intensity of the emotion he or she was feeling whilst speaking about each event (using the same scale that the participants used (from one = no emotion to nine =very strong emotion)). This differs slightly from the procedure used by Mackes et al. (2018), in which each clip was filmed and then rated separately before proceeding to the next clip. It appears unlikely that this slight change in procedure substantially impacted on the validity of the narrator’s ratings of emotional intensity, as in both cases the narrator was watching the clip they had previously recorded.

The narrator was paid £20 per hour for her time.

**Description of EAT Film Clips**

For the ‘happy’ film clip, the male narrator described a memory of going skateboarding with his friends. The female narrator discussed feeding elephants whilst on holiday. For the ‘angry’ film clip, the male narrator talked about a time when his bike was stolen; the female narrator described an incident when she was unfairly told off by a teacher at school. For the ‘sad’ film clip, the male narrator recalled a telephone conversation with his aunt, who was considering stopping her cancer treatment as it was making her so unwell. The ‘sad’ event described by the female narrator was her dog suddenly dying. For the ‘frightened’ film clip, the male narrator talked about staying overnight in a cottage and hearing strange noises whilst trying to sleep. The frightening event described by the female narrator was going bungee jumping, and having second thoughts about whether to jump.

A different female narrator was used in the old film clips. For ‘Happy’ she described finding out that she had got into her chosen university; for ‘Sad’ she discussed getting a rejection letter from a university; for ‘Angry’ she described arguing with her housemate about whether her friend could move into their spare room. For ‘frightened’, she described a domestic violence incident (using this clip raised some ethical concerns, which is partly why new clips were filmed with another female narrator).

For the ‘neutral’ film clips, all narrators described their bedroom.

**Supplementary Results**

**Trait Empathy (IRI Findings)**

*

*

*Supplementary Figure 1*. A bar chart showing the self-reported empathy scores in the ASD and TD Groups. IRI, Interpersonal Reactivity Index.

**EA Task: Empathic Accuracy**

Supplementary Table 1

*EA Scores (Including Participants who watched the original film clips)*

| EA Score | ASD Group  (n= 29) | TD Group  (n= 30) | *p* Value | Effect Size (*r*) |
| --- | --- | --- | --- | --- |
| Total 1.03 1.2  Happiness 1.03 1.05  Sadness 1.22 1.39  Anger 0.93 1.20  Fear 1.00 1.16 | | | .012*  .439  .030  <.001*  .077 | -0.33  -0.10  -0.28  -0.52  -0.23 |

The ASD Group’s Total EA Score was significantly lower than the TD group’s score, *U=* 259, z= -2,5, *p=* .012, *r=* -0.33 (see Supplementary Table 1). The ASD group also scored significantly lower in response to the clips displaying Anger compared with the TD group, *U=* 175, *z=* -3.94, *p*= <.001, *r=* -0.52. Furthermore, the ASD group scored slightly, but not significantly, lower than the TD group in response to clips displaying Sadness (*U=* 292, *z=* -2.168, *p=* .30) and Fear (*U=* 306.5, *z=* -1.766, *p=* .077).

**Correlations between Alexithymia and Empathy**

*Supplementary Figure 2.* Scatter plot (with trendline and 95% confidence intervals) to show the significant positive correlation between Alexithymia (TAS-20) and Interpersonal Reactivity Index (IRI) Personal Distress scores in the ASD Group.

Supplementary Table 2.

*Correlations between Alexithymia and EAT Scores in the ASD Group*

|  | **Correlation**  **Coefficient (*r*)** | **Significance**  **Level (*p*)** |
| --- | --- | --- |
| **Cognitive Empathy** |  |  |
| Total CE | .053 | .785 |
| Happiness | -.142 | .464 |
| Sadness | .152 | .430 |
| Anger | .366 | .051 |
| Fear | -.136 | .481 |
| Neutral | -.033 | .866 |
| **Affective Empathy** |  |  |
| Total AE | -.026 | .892 |
| Happiness | -.357 | .057 |
| Sadness | .155 | .422 |
| Anger | .108 | .575 |
| Fear | .050 | .795 |
| **Empathic Accuracy^1^** |  |  |
| Total EA | .011 | .955 |
| Happiness | -.041 | .855 |
| Sadness | .062 | .785 |
| Anger | -.041 | .858 |
| Fear | -.046 | .842 |

**^1^**This analysis only included participants who watched the new video clips.

|  | **Correlation**  **Coefficient (*r*)** | **Significance**  **Level (*p*)** |
| --- | --- | --- |
| **Cognitive Empathy** |  |  |
| Total CE | -.045 | .815 |
| Happiness | -.065 | .732 |
| Sadness | .306 | .100 |
| Anger | -.185 | .327 |
| Fear | -.009 | .962 |
| Neutral | -.025 | .895 |
| **Affective Empathy** |  |  |
| Total AE | -.173 | .361 |
| Happiness | -.350 | .058 |
| Sadness | .059 | .756 |
| Anger | -.085 | .655 |
| Fear | -.275 | .142 |
| **Empathic Accuracy** |  |  |
| Total EA | .087 | .647 |
| Happiness | .098 | .608 |
| Sadness | -.132 | .488 |
| Anger | .142 | .453 |
| Fear | .066 | .729 |

Supplementary Table 3.

*Correlations between Alexithymia and EAT Scores in the TD Group*

*Supplementary Figure 3.* A scatterplot (with trendline and 95% confidence intervals) showing the relationship between alexithymia and Total CE score on the EAT in the ASD group.

*Supplementary Figure 4.* A scatterplot (with trendline and 95% confidence intervals) showing the relationship between alexithymia and Total CE score on the EAT in the TD group.

*Supplementary Figure 5.* A scatterplot (with trendline and 95% confidence intervals) showing the relationship between alexithymia and Total AE score on the EAT in the ASD group.

*Supplementary Figure 6.* A scatterplot (with trendline and 95% confidence intervals) showing the relationship between alexithymia and Total AE score on the EAT in the TD group.

**Correlations between IRI, EA, AE and CE scores**

To check for criterion validity, the IRI scores were correlated with the EAT scores. Total AE scores were significantly positively correlated with Total IRI scores (r(58)= .359, *p=* .005), and with Empathic Concern (*r*(58)= .449, *p*=<.001) and Perspective Taking (*r*(58)= .451, *p= <.*001) subscale scores. Correlations between Total AE score and scores on the Personal distress and Fantasy subscales were non-significant (*r*(58)=.122, *p*=.355 for Personal Distress; *r*(58)=-.063, *p*=.633 for Fantasy).

Total EA Scores were not significantly correlated with Total IRI scores (*r*(58)=.165, *p*=.215, and no significant correlations were found between Total EA and any of the individual IRI scores. Similarly, no significant correlations were found between Total CE score and Total IRI scores (*r*(58)=.142, *p*=.284, or or any of the individual IRI scores.

**Relationship between Anxiety and Empathy Deficits in the ASD Group**

Mann Whitney *U* tests were used to explore whether empathy deficits in the ASD group could be explained by the presence of anxiety or social anxiety. Eleven participants in the ASD group and four participants in the TD Group reported current anxiety. Seven participants in the ASD group and zero participants in the TD group reported current social anxiety. Analysing the new clips only, EA scores for anger did not differ according to whether participants in the ASD group reported currently experiencing anxiety (*U= 44.0, z*= -.488, *p*= .733) or social anxiety (*U=* 35.0, *z*= -.959, *p=* .338). No significant differences were found for IRI Empathic Concern when comparing those who reported currently experiencing anxiety (U= 73.50, z= -1.151, p= .250) or social anxiety (U= 63.0, z= -.716, p= .500). There was a marginally significant difference in IRI Perspective Taking scores, with those reported current Anxiety scoring slightly higher than those who were not experiencing anxiety (*U*= 55.00, *z*= -1.99, *p*= .047). The same trend was found in the TD group, although the differences between those who did and did not report anxiety were not statistically significant. Participants with ASD who reported experiencing current social anxiety scored significantly higher on IRI Perspective Taking compared to those who did not report current social anxiety (*U*= 28.0, *z*= -2.513, *p*= .012).

In light of these findings, Mann-Whitney U tests were re-run to tests for differences in IRI perspective taking across the TD Group and ASD Group, excluding those who reported current social anxiety in the ASD Group. The TD group still scored significantly higher on Perspective Taking (*U*= 124.50, *z*= -3.196, *p*= .000). Similarly, when participants in the ASD Group who reported current anxiety were excluded, scores on Perspective Taking remained significantly higher in the TD Group (*U*= 102.50, *z*= -3.668, *p*= .000).
